# Genetic Phenotypes of Alzheimer’s Disease: Mechanisms and Potential Therapy

**DOI:** 10.1007/s43657-023-00098-x

**Published:** 2023-04-03

**Authors:** Meina Quan, Shuman Cao, Qi Wang, Shiyuan Wang, Jianping Jia

**Affiliations:** 1 Innovation Center for Neurological Disorders and Department of Neurology, Xuanwu Hospital, Capital Medical University, Beijing, 100053 China; 2 National Medical Center for Neurological Disorders and National Clinical Research Center for Geriatric Diseases, Beijing, 100053 China; 3 Beijing Key Laboratory of Geriatric Cognitive Disorders, Beijing, 100053 China; 4 Clinical Center for Neurodegenerative Disease and Memory Impairment, Capital Medical University, Beijing, 100053 China; 5 Center of Alzheimer’s Disease, Collaborative Innovation Center for Brain Disorders, Beijing Institute of Brain Disorders, Capital Medical University, Beijing, 100053 China; 6 Key Laboratory of Neurodegenerative Diseases, Ministry of Education, Beijing, 100053 China

**Keywords:** Alzheimer's disease, Genetic phenotypes, Molecular mechanism, Gene therapy

## Abstract

Years of intensive research has brought us extensive knowledge on the genetic and molecular factors involved in Alzheimer's disease (AD). In addition to the mutations in the three main causative genes of familial AD (FAD) including *presenilins* and *amyloid precursor protein* genes, studies have identified several genes as the most plausible genes for the onset and progression of FAD, such as *triggering receptor expressed on myeloid cells 2*, *sortilin-related receptor 1*, and *adenosine triphosphate-binding cassette transporter subfamily A member 7*. The *apolipoprotein E ε4* allele is reported to be the strongest genetic risk factor for sporadic AD (SAD), and it also plays an important role in FAD. Here, we reviewed recent developments in genetic and molecular studies that contributed to the understanding of the genetic phenotypes of FAD and compared them with SAD. We further reviewed the advancements in AD gene therapy and discussed the future perspectives based on the genetic phenotypes.

## Introduction

Alzheimer's disease (AD) is a neurodegenerative disease that is biologically defined by the presence of β-amyloid-containing plaques and tau-containing neurofibrillary tangles (NFT). After years of intensive research, we have gained extensive knowledge of the genetic factors and their mechanisms in AD. Genetically, AD can be categorized as sporadic AD (SAD) and familial AD (FAD) based on family history (Jia et al. 2020b). FAD accounts for 15–25% of total AD and has presented a useful model in studying the pathogenesis and trajectory of the disorder's progress (Goldman et al. 2011).

AD is affected by multiple genes, which can be further divided into pathogenic genes and risk genes. Known AD pathogenic genes include *presenilin 1* (*PSEN1*), *presenilin 2* (*PSEN2*), and *amyloid precursor protein* (*APP*). These types of genes mainly cause early onset AD (EOAD), accounting for about 1% of all AD patients (Goate et al. 1991; Levy-Lahad et al. 1995; Sherrington et al. 1995). *Apolipoprotein E* (*APOE*) *ε4* is a widely confirmed risk gene for SAD, usually late-onset AD (LOAD), accounting for about 50% of this type of patients (Strittmatter et al. 1993; Coon et al. 2007). In SAD, an *APOE ε4* allele can increase the risk of AD by about three times, while two *APOE ε4* alleles can increase the risk of AD by approximately 12 times (Liu et al. 2013; Jia et al. 2020c). Interestingly, recent large cohort studies also found that the genetic risk effect of *APOE ε4* are higher in FAD with unknown mutation than in SAD (Jia et al. 2020c).

In addition to the three major pathogenic genes and *APOE ε4*, genome-wide association studies (GWAS) have revealed a large number of AD susceptibility loci, while whole genome sequencing (WGS) and whole exome sequencing (WES) studies have identified many AD-associated rare variants. These variants are enriched in *triggering receptor expressed on myeloid cells 2* (*TREM2*), *sortilin-related receptor 1* (*SORL1*), *adenosine triphosphate-binding cassette transporter subfamily A member 7* (*ABCA7*), *complement receptor 1* (*CR1*), *cluster of differentiation 33* (*CD33*), *clusterin* (*CLU*), *bridging integrator 1* (*BIN1*), and *death-associated protein kinase 1* (*DAPK1*) (Li et al. 2006, 2021; Rogaeva et al. 2007; Beecham et al. 2009; Carrasquillo et al. 2009; Bellenguez et al. 2022; Jack 2022). Many of them have been verified in FAD population. For example, the rare variant *TREM2* G145T was present in several members of a family with probable AD-type dementia without the three known pathogenic variants (Karsak et al. 2020). Some rare *SORL1* variants are reported in FAD pedigrees (Gomez-Tortosa et al. 2018). In 77.3% of *ABCA7* carriers' families, there were AD patients (Bossaerts et al. 2021).

Here, we reviewed recent advances in genetic studies that have contributed to the understanding of AD pathogenesis. We summarized the genetic and molecular mechanisms involved such as the amyloid cascade hypothesis, tau-dependent pathology, synaptic dysfunction, neuro-inflammation and oxidative stress, and lipid metabolism. We further compared the pathogenesis between FAD and SAD and reviewed preclinical and clinical studies of AD gene therapy. Such integration is not only helpful for understanding the commonality and heterogeneity in pathogenesis, but also conducive to clinical diagnosis and classification, development of gene-targeted therapies, and design of clinical trials based on different genetic phenotypes.

## Pathogenic Genes for FAD

**Fig. 1:**
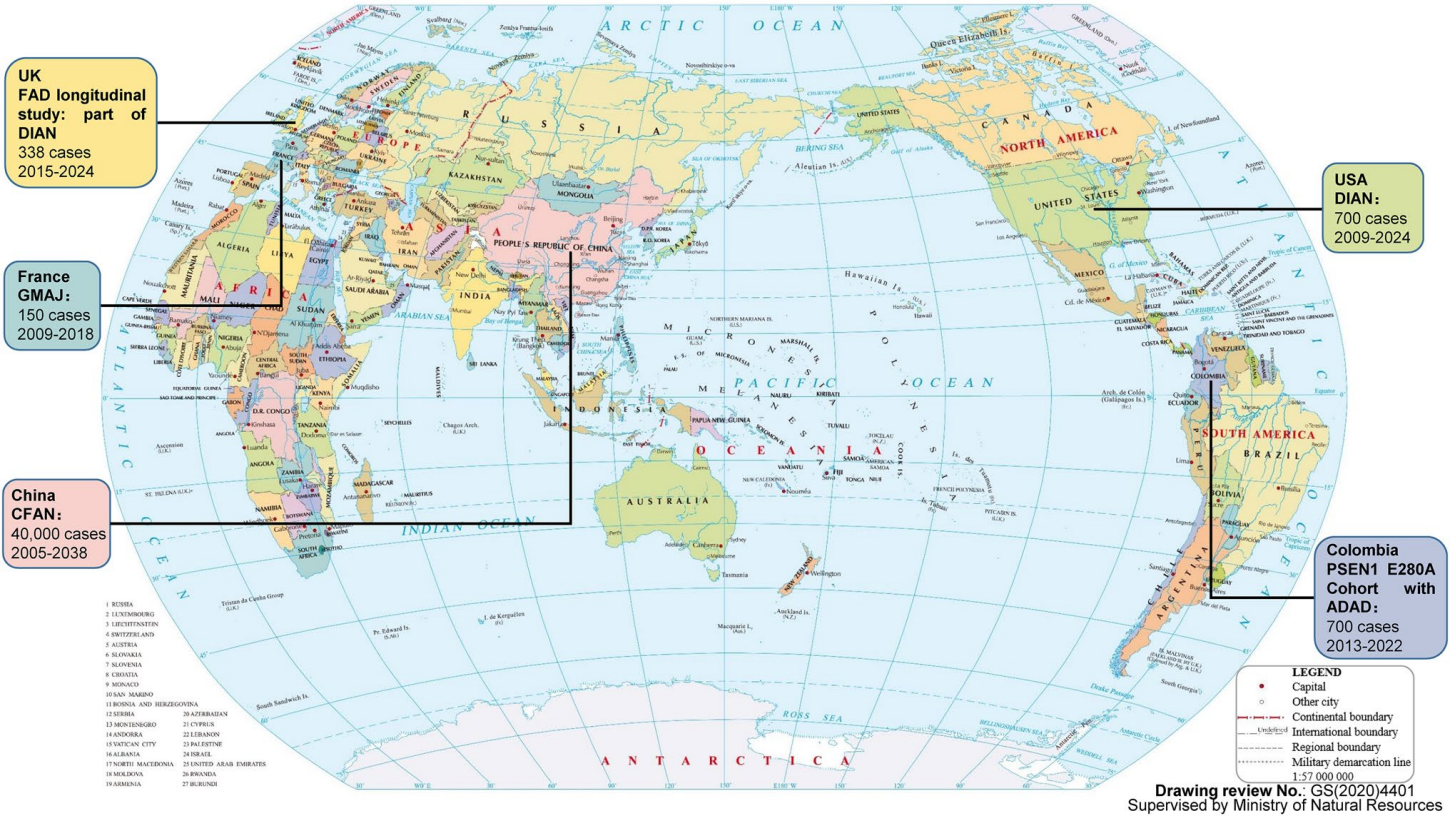
Major FAD cohort studies in the world. FAD research is mainly concentrated in the United States of America, United Kingdom, Colombia, France, and China, gradually forming a situation of international multicenter cooperation. DIAN: Dominantly inherited Alzheimer network; CFAN: Chinese familial Alzheimer's network; GMAJ: Genetics of Mendelian forms of young onset AD; ADAD: Autosomal-dominant AD. Reproduced from the map of the world, which was downloaded from the website (http://bzdt.ch.mnr.gov.cn/index.html), with drawing review No.: GS(2020)4401, and supervised by Ministry of Natural Resources of China

There are several large genetic cohort studies of FAD in the world (Fig. 1). FAD research is mainly concentrated in the United States of America (Bateman et al. 2012; Chhatwal et al. 2022), United Kingdom (Oxtoby et al. 2018; Weston et al. 2018), Colombia (Ramirez Aguilar et al. 2019; Quiroz et al. 2020), France (Rovelet-Lecrux et al. 2012; Zarea et al. 2016), and China (Jia et al. 2005, 2020b; Quan et al. 2020), and gradually forming multi-center collaboration. The most representative FAD study is the Dominantly Inherited Alzheimer Network (DIAN) study in the United States of America, which found many AD genetic and diagnostic biomarkers (Bateman et al. 2012; Chhatwal et al. 2022). The largest FAD cohort study is the Chinese familial Alzheimer's Network (CFAN), aiming to recruit 40,000 subjects in FAD (clinicaltrials.gov registration ID: NCT03657732). From genetic cohort studies, three main causative genes of FAD including *PSEN1*, *PSEN2*, and *APP*, and many associated variants were reported. For example, *PSEN1* E280A (glutamic acid-to-alanine mutation at codon 280) variant was reported from Colombia kindred (Lopera et al. 1997), which further initiated the Colombia *PSEN1* E280A cohort of autosomal dominant Alzheimer's disease (ADAD). *PSEN*1 V97L mutation was reported from Chinese families (Jia et al. 2005), which further initiated the CFAN cohort.

### *APP*

APP is a transmembrane protein widely expressed in the central nervous system and peripheral tissues. Proteolytic cleavage of APP generates the Aβ peptide, which aggregates into plaques, is one of the major hallmarks of AD (Tackenberg et al. 2020). Amyloid-β protein precursor (AβPP) can be cleaved by proteases in canonical and non-canonical pathways. In the canonical pathway, AβPP is cut by α-secretase, producing a soluble APPα peptide and α-C-terminal fragment which can be further cleaved by γ-secretase, generating APP intracellular domain (AICD) and a non-pathogenic three kDa product (Guo et al. 2021). In non-canonical pathway, AβPP is cut by β-secretase, producing a soluble APPβ peptide and β-C-terminal fragment which can be further cleaved by γ-secretase, generating Aβ48 or Aβ49 and AICD. Aβ48 or Aβ49 continued to produce Aβ45, Aβ42, Aβ38 or Aβ46, Aβ43, and Aβ40, respectively, under the action of γ-secretase (Andrew et al. 2016). The anomalous processing of APP leads to the production of Aβ40 and Aβ42 monomers, which further oligomerize and aggregate into senile plaques in AD (Zou et al. 2007; Tiwari et al. 2019). *APP* V717I was the first gene mutation found to be linked with inherited AD, which could influence the stability of Aβ deposition, alter translational regulation at the mRNA level of this protein, or increase long Aβ secretion to foster amyloid deposition (Goate et al. 1991; Almqvist et al. 1993; Suzuki et al. 1994). Subsequent studies have identified more *APP* mutations, all of which contribute to FAD. Interestingly, most of these mutations found in *APP* are located in exons 16 and 17 on chromosome 21, near the α-secretase cleavage site, in the central part of the Aβ peptide or near the γ-secretase site of the attack, giving rise to an increase or alteration in Aβ production (Aβ 1–42 fragment) (Theuns et al. 2006; Tian et al. 2010; Piaceri et al. 2013), or alteration of Aβ42:40 ratio (Tian et al. 2010). The Australian *APP* L723P mutation causes local unfolding of the C-terminal turn of the APP transmembrane domain helix, and increases its accessibility to water required for cleavage of the protein backbone by γ-secretase in the ε-site, resulting in accumulation of the pathogenic forms of Aβ (Bocharov et al. 2019). The Swedish *APP* K670N/M671L mutation in exon 16 occurs at the amino terminal of Aβ is proximal to the β-secretase cleavage site, and it increases the production of total Aβ through dramatically enhancing β-secretase cleavage of APP17 (Mullan et al. 1992; Vassar et al. 1999). Osaka mutation (*APP* E693Δ) is the deletion of codon 693 of *APP* gene, resulting in mutant Aβ lacking the 22nd glutamate, which accelerates Aβ oligomerization without forming amyloid fibrils and disrupts synaptic function to cause cognitive impairment in humans (Tomiyama et al. 2020).

Aβ can promote tau pathology, and its toxicity is also tau-dependent (Gotz et al. 2008). Aβ alone does not cause neurodegeneration but induces toxicity through the phosphorylation of wild-type tau in an N-methyl-D-aspartate (NMDA) receptor-dependent pathway (Tackenberg et al. 2009). APP is involved in several neuroplasticity-signaling pathways, such as NMDA-protein kinase A (PKA)-cyclic adenosine monophosphate response element binding protein (CREB)-brain-derived neurotrophic factor (BDNF), reelin, wingless, and notch (Forero et al. 2006). Hippocampal accumulation of mutant *APP* and Aβ is responsible for abnormal mitochondrial dynamics and defective biogenesis, reduced microtubule-associated protein 2 (MAP2), autophagy, mitophagy, synaptic proteins and dendritic spines, and changes in mitochondrial structure and function, leading to neuronal dysfunction and impaired hippocampal-based learning and memory (Manczak et al. 2018; Reddy et al. 2018). In a novel *APP* knock-in mouse model (*APP* Swedish, Arctic and Austrian), fibrillar Aβ in microglia is associated with lipid dyshomeostasis, which is consistent with lysosomal dysfunction and foam cell phenotypes as well as profound immuno-metabolic perturbations (Xia et al. 2022). A rat model with three *APP* mutations and humanized Aβ sequence knocked into the rat's *APP* gene exhibited pathologies and disease progression resembling those in human patients. Specifically, Aβ plaques were deposited in relevant brain regions, and other mechanisms were found, including microglia activation and gliosis, progressive synaptic degeneration, tau pathology, neuronal apoptosis and necroptosis, brain atrophy, and AD-relevant cognitive deficits (Pang et al. 2022).

### *PSEN1*

PSEN1 serves as a catalytic subunit of γ-secretase complex, which mediates the proteolytic liberation of Aβ from AβPP. PSEN1 is also involved in non-proteolytic functions such as protein trafficking, regulation of ion channel, cholesterol metabolism, and homeostatic synaptic scaling (Li et al. 2000; Pratt et al. 2011; Cho et al. 2019). *PSEN1* mutation leads to the production of longer amyloidogenic Aβ peptides and increased Aβ42:40 ratio (Selkoe 2001; Fernandez et al. 2014), causing the most aggressive form of inherited AD. *PSEN1* mutation carriers with an earlier age of onset and considerable phenotypic variability show mutation-specific effects and a trend towards a reduced abundance of newborn neurons, supporting a premature aging phenotype and altered neurogenesis (Arber et al. 2021). *PSEN1* mutants potentiate cell cycle arrest and apoptosis, and the degree to which the different mutants inhibit cell cycle progression correlates with the age of onset (Janicki et al. 2000). *PSEN*1 S169del mutation altered APP processing and Aβ generation, and promoted senile plaque formation as well as learning and memory deficits in mice (Zhang et al. 2020a). *PSEN*1 V97L mutation induced self-replication of Aβ oligomers (AβO) in astrocytes and triggered neuronal injury in mice (Wang et al. 2019).

Other pathogenic mechanisms are also reported in *PSEN1* mutation models. In the *PSEN1* ΔE9 cells, the elevated cellular cholesterol level contributes to the altered APP processing by increasing APP localized in lipid rafts (Cho et al. 2019). In primary fibroblasts from patients bearing *PSEN1* mutations, Aβ42 oligomers are recruited to lipid rafts, resulting in lipid peroxidation, calcium dyshomeostasis and membrane permeabilization, and amyloid toxicity (Evangelisti et al. 2013). Primary hippocampal neurons from *PSEN1* transgenic mice exhibit increased production of Aβ peptide 42/43 and vulnerability to excitotoxicity in a gene dosage-dependent manner. Neurons expressing mutant *PSEN1* exhibit enhanced calcium responses to glutamate increased oxyradical production and mitochondrial dysfunction (Guo et al. 1999). *PSEN1* mutations also increase oxidative stress and perturb calcium signaling in lymphocytes in ways that alter their production of inflammatory cytokines that are critical for proper immune responses (Mattson 2002; Schuessel et al. 2006). Inflammatory cytokines, such as tumor necrosis factor-α (TNFα), interleukin (IL)-1α, IL-1β, IL-1 receptor antagonist, and IL-6, are significantly greater in the hippocampus and cerebral cortex of *PSEN1* mutant mice as compared to wild-type mice (Lee et al. 2002).

### *PSEN2*

PSEN2 forms the catalytic core of the γ-secretase complex, a function shared with its homolog PSEN1, which is ultimately responsible for Aβ formation (Pizzo et al. 2020). Besides its enzymatic activity, PSEN2 is a multifunctional protein, which is specifically involved in the modulation of several cellular processes, such as proinflammatory response, mitochondrial function, ubiquilin, and autophagy (Pizzo et al. 2020). AD-causing mutations shift Aβ length by destabilizing γ-secretase-Aβ interactions, which is fundamental to the disease (Szaruga et al. 2017, 2021).

*PSEN2* mutations either increase Aβ production or alter the Aβ42/40 ratio that contributes to the development of AD (Loy et al. 2014; Pang et al. 2021). *PSEN2* N141I mutation produces an AD phenotype with a wide range of onset ages overlapping both EOAD and LOAD, often associated with seizures, rapidly progressive dementia, neurologic and behavioral symptoms, high penetrance and typical AD neuropathology (Jayadev et al. 2010; Muchnik et al. 2015). PSEN2 participates in maintaining the basal and cytokine-induced expression of the innate immunity regulating microRNA, and its dysfunction or deficiency could result in disrupted innate immune homeostasis and unchecked proinflammatory activation (Jayadev et al. 2013; Fung et al. 2020). AD-linked *PSEN2* mutants alter multiple Ca^2+^ pathways and the functional consequences of this Ca^2+^ dysregulation in AD pathogenesis (Greotti et al. 2019; Galla et al. 2020). They decrease the Ca^2+^ content of the endoplasmic reticulum (ER), modulate Ca^2+^ shuttling between the ER and mitochondria, and reinforce ER-mitochondria tethering (Zampese et al. 2011; Rossini et al. 2021). *PSEN2* knock-out neurons show a marked reduction in ER-mitochondria apposition and a slight alteration in mitochondrial respiration (Rossi et al. 2021). *PSEN2* mutation also actions on autophagy, depending on its ability to partially deplete ER Ca^2+^ content and reduce cytosolic Ca^2+^ response upon inositol trisphosphate-linked cell stimulations (Fedeli et al. 2019). Overexpression of *PSEN2* N141I mutation causes cell starvation and cell death, and ubiquilin expression protects cells against starvation by modulating biogenesis and endoproteolysis of PSEN2 proteins (Rothenberg et al. 2010). *PSEN2* mutation is also involved in the abnormalities of lipid profile, where the levels of cholesterol, low-density lipoprotein and triglyceride are increased, but the level of high-density lipoprotein is decreased (Nguyen et al. 2006).

## *APOE ε4* and Other Risk Genes in FAD

Over 130 AD-associated susceptibility loci have been identified by GWAS, while WGS and WES studies have identified AD-associated rare variants. Except for *APOE*, these variants are enriched in *TREM2, SORL1, ABCA7, CR1, CD33, CLU, BIN1*, and more genes, but with smaller effect size, lower population prevalence, or both compared with *APOE ε4* (Li et al. 2021; Bellenguez et al. 2022; Jack 2022). Studies have identified several genes as the most plausible genes for FAD, including *TREM2, SORL1*, and *ABCA7* (Campion et al. 2019; Scheltens et al. 2021).

### *APOE*

APOE is a lipoprotein that is expressed in the brain, liver, and myeloid cells, and it is involved in cholesterol and lipid transportation, neuronal growth, and immune-regulation. Three different alleles of *APOE* encode three isoforms, including *APOE ε2*, *APOE ε3*, and *APOE ε4* (Poirier et al. 1993). Although the three isoforms differ by only two amino acids, the structure and function of *APOE* isoforms are significantly altered (Neuner et al. 2020). The *APOE ε4* allele is the strongest genetic risk factor for AD. One copy of the ε4 allele increases the risk of AD by two to six times, and the presence of two copies increases the risk by 7.2 to 21.8 times (Genin et al. 2011; Jia et al. 2020c; Qin et al. 2021). It is widely accepted that carrying the *APOE ε4* allele reduces the age of onset by about 12 years (Corder et al. 1993; Belloy et al. 2019). Since its identification, *APOE ε4* allele has been regarded as a risk factor for SAD instead of FAD, because it is neither necessary nor sufficient to cause AD (Cacace et al. 2016), and its inheritance does not follow an autosomal dominant pattern such as *APP*, *PSEN1*, and *PSEN2* mutations (van Duijn et al. 1994; Frisoni et al. 2022). However, studies indicate that *APOE ε4* also plays an important role in FAD. Actually, *APOE ε4* was first identified and shown to be associated with the increased risk of AD in late-onset FAD, and then association studies in cohorts identified it as a major genetic risk factor for late-onset SAD (Pericak-Vance et al. 1991; Corder et al. 1993; Strittmatter et al. 1993). Subsequently, a study demonstrated a significant association between *APOE ε4* and EOAD which is modified by a family history of dementia. Among patients, the *APOE ε4* allele frequency was 1.6 times higher in those with positive family history than in those without (van Duijn et al. 1994). In spite of this, they think the *APOE ε4* allele cannot fully explain familial aggregation of EOAD as among *APOE ε4* carriers as well as non-carriers the risk of EOAD increased significantly for those with a positive family history of dementia (van Duijn et al. 1994).

However, a recent study in a cohort of 404 Chinese pedigrees with FAD showed different results. They found that among patients without *PSENs*/*APP* mutations, 44.31% carried one *APOE ε4* allele, while 14.85% carried two *APOE ε4* alleles (Jia et al. 2020b). These percentages were much higher than those in SAD patients. Furthermore, patients with two ε4 alleles are more likely to develop FAD than those with a single ε4 allele and other subtypes of AD, indicating that increased *APOE ε4* gene dosage may promote the development of FAD (Jia et al. 2020c). This phenomenon called the *APOE ε4* diploid enhancement of familial aggregation has been reported in other studies, suggesting that *APOE ε4* plays an important role in familial aggregation (Martinez et al. 1998; Huang et al. 2004). These results urge a reappraisal of the impact of *APOE ε4* in FAD. In addition, some studies in FAD suggest that *APOE ε4* influences the age at which AD occurs, where onset age decreases in presence of the ε4 allele (Velez et al. 2016; Reyes-Dumeyer et al. 2022). Another study showed that at the age of 85, the lifetime risk of AD without reference to *APOE* genotype was 11% to 14% for male and female, respectively, while the risk ranged from 51% to 60% for *APOE ε4/ε4* carriers, and from 23% to 30% for *APOE ε3/ε4* carriers, which is consistent with semi-dominant inheritance of a moderately penetrant gene (Genin et al. 2011).

*APOE ε4* negatively impacts a plethora of biological processes associated with AD in human patients. Namely, *APOE ε4* accelerates neurodegeneration and cognitive deficits; increases Aβ deposition by promoting its production and fibrillization and impairing degradation/clearance pathways; increases the accumulation of tau pathology by increasing its phosphorylation and fibrillization, and accelerating its spread; amplifies gliosis and inflammation by exacerbating neuroinflammatory response, impairing astrocytes ability to maintain synapses, increasing neurons phagocytosis and decreasing toxic proteins removal; disrupts network activity and functional connectivity within or between brain regions; and reduces central nervous system glucose metabolism (Koutsodendris et al. 2022). Other pathogenic mechanisms include lipid metabolism, neuronal signaling, mitochondrial function, and blood–brain barrier (Long et al. 2019; Serrano-Pozo et al. 2021; Jackson et al. 2022; Koutsodendris et al. 2022; Martens et al. 2022). It is possible that *APOE ε4*-induced detrimental effects could work independently or in concert with one another. Of note, the precise mechanism by which *APOE ε4* increases AD risk remains inconclusive, so further investigation of the *APOE* gene is critical for developing therapeutics (Koutsodendris et al. 2022).

### *TREM2*

TREM2 is a single-pass transmembrane receptor of the immunoglobulin superfamily that was initially identified in monocyte-derived dendritic cells and mouse macrophages (Ulland et al. 2018). TREM2 is a receptor for Aβ that mediates microglial function, including proliferation, survival, clustering, and phagocytosis (Ulland et al. 2017; Zhao et al. 2018). It is essential for microglia-mediated synaptic refinement during the early stages of brain development (Filipello et al. 2018). TREM2 promotes the optimal microglial function required to attenuate AD progression, enabling microglial progression to a fully mature disease-associated microglia profile and ultimately sustaining the microglial response to Aβ plaque-induced pathology (Ulland et al. 2018).

The minor allele frequency of R47H in the *TREM2* gene was much lower while the effect size was as high as *APOE ε4* (Guerreiro et al. 2013; Jonsson et al. 2013). The association of R47H with elevated LOAD risk was successfully replicated in European-American, Spanish, French-Caucasian, North American-Caucasian and African-American populations, but failed in Han Chinese population (Carmona et al. 2018). In *TREM2* R47H carriers, the role of TREM2 receptor in the microglial clearance of aggregation-prone proteins is compromised (Korvatska et al. 2015). *TREM2* R47H mutation AD also demonstrates upregulation of interferon type I response and pro-inflammatory cytokines accompanied by induction of natural killer group 2 member D (NKG2D) stress ligands (Korvatska et al. 2020). It induces and exacerbates tau-mediated spatial memory deficits in female mice (Sayed et al. 2021). Furthermore, transcriptomic changes from these mice had substantial overlaps with *TREM2* R47H microglia in human AD brains, including robust increases in proinflammatory cytokines, activation of AKT signaling, and elevation of a subset of disease-associated microglia signatures (Sayed et al. 2021). In a family with probable AD-type dementia without the three known pathogenic variants, another rare variant *TREM2* G145T was present in severely affected, putatively affected, and unaffected members, suggesting incomplete penetrance and variable age of onset. This variant led to intrinsically disordered region shortening and structural changes of TREM2, resulting in an impairment of cellular responses upon receptor activation (Karsak et al. 2020). The absence of *TREM2* resulted in repetitive behavior and altered sociability in mice, impaired synapse elimination, enhanced excitatory neurotransmission, and reduced long-range functional connectivity (Filipello et al. 2018). Deleting *TREM2* exacerbated tau accumulation and spreading, and promoted brain atrophy only if Aβ pathology is present, indicating that *TREM2* may slow AD progression and reduce tau-driven neurodegeneration by restricting the degree to which Aβ facilitates the spreading of pathogenic tau (Lee et al. 2021).

### *SORL1*

The *SORL1* gene is a regulator of endosomal traffic and recycling in human neurons. *SORL1* encodes sorting-related receptor with A-type repeats (SORLA), a key protein involved in APP processing and the secretion of Aβ peptide (Campion et al. 2019). Some rare *SORL1* variants are reported in FAD pedigrees, supporting the putative autosomal dominant inheritance and cause of EOAD (Gomez-Tortosa et al. 2018). These variants include *SORL1* T588I change, T2134 alteration, Trp848Ter, Gly1871Val, Glu270Lys, Gly852Ala, Arg1702Met, Asn1809Ser, Asp2065Val, Ala2173Thr, a splice-site variant (chromosome position 121,466,486 G > A), Arg1303Cys, c.3050-2A > G, c.5195G > C, V1482fs (Pottier et al. 2012; Thonberg et al. 2017). Depletion of *SORL1* significantly impacts the endosomal recycling pathway in neurons for APP and glutamate receptor subunit α-Amino-3-hydroxy-5-methyl-4-isoxazolepropionic acid (GLUA1) at the level of the recycling endosome and trafficking to the cell surface, conversely, increased *SORL1* expression enhances endosomal recycling for APP and GLUA1 (Mishra et al. 2022b). Truncating mutation of *SORL1* results in mitochondrial dysfunction and enlarged endosomes in human neurons due to *SORL1* haploinsufficiency, while complete loss of *SORL1* leads to additional defects in lysosome function and autophagy (Barthelson et al. 2020; Hung et al. 2021). A study of cortices and hippocampus of *SORL1*-deficient mice showed increased synapsin 1 and 2, however, the specific role of *SORL1* in synaptic function in FAD remains unclear (Hartl et al. 2016; Perdigao et al. 2020). There are also LOAD cases with rare *SORL1* variants, such as *SORL1* A528T, T947M, and A674S. Functionally, the variants impair SORL1 protein function and weaken its interaction with full-length APP, altering levels of Aβ and interfering with APP trafficking (Cuccaro et al. 2016; Louwersheimer et al. 2017).

### *ABCA7*

There were AD patients in 77.3% of the families of *ABCA7* carriers, suggesting a positive family history of the disease (Bossaerts et al. 2021). In a Belgian AD cohort, 22 affected members carried an *ABCA7* E709fs. All carriers except one presented with memory complaints (Van den Bossche et al. 2016). Two rare *ABCA7* variants (rs143718918 and rs538591288) were identified in two independent German AD families, respectively. The rs143718918 variant causes a missense mutation, and the rs538591288 deletion causes a frameshift mutation of *ABCA7* (May et al. 2018). *ABCA7* heterozygous variant c.3706C > T p.(Avg 1236Cys) was found in seven affected members in a Saudi family, which is likely pathogenic because of the presenting complex neurological disease due to decreased clearance of Aβ and α-synuclein (Algahtani et al. 2020). Missense variants in *ABCA7* (P143S and A1507T) were significantly associated with FAD when compared with the East Asian controls in the ExAC database (Zhang et al. 2020b). *ABCA7* rs376824416 3’-UTR splice was identified in four siblings of one family in a non-Hispanic White and African-American cohort, which was nominally associated with LOAD (Kunkle et al. 2017). A missense mutation in *ABCA7* G1820S co-segregated with AD in one pedigree, which induced protein mislocalization and resulted in a lack of functional protein at the plasma membrane (Bossaerts et al. 2022).

ABCA7 belongs to the “A” subfamily of the adenosine triphosphate-binding cassette transporters. ABCA7 deficiency results in accelerated Aβ production, likely by facilitating endocytosis and/or processing of APP (Aikawa et al. 2018). While ABCA7 has been shown to mediate phagocytic activity in macrophages, it is also involved in the microglial Aβ clearance pathway (Abe-Dohmae et al. 2021). *ABCA7* loss of function may contribute to AD pathogenesis by altering proper microglial responses to acute inflammatory challenges during the development of amyloid pathology (Aikawa et al. 2019). ABCA7 also regulates brain fatty acid metabolism during lipopolysaccharide-induced acute inflammation (Aikawa et al. 2021). *ABCA7*-deficient mice's brain had significantly lower levels of several sphingomyelin (SM) species with long-chain fatty acids, and anomalies in synaptic plasticity in the synapse of the lateral entorhinal cortex, that were rescued by extracellular SM supplementation (Iqbal et al. 2022).

Table 1 summarized the mechanisms of the major pathogenic and risk genes for AD.

**Table 1:** The mechanisms of major pathogenic and risk genes for FAD

Gene	Amyloid cascade hypothesis	Tau-dependent pathology	Synaptic dysfunction	Neuro-inflammation, oxidative stress and immune dysfunction	Lipid metabolism	Mitochondrial dysfunction and energy metabolism	Autophagy	Other pathogenic mechanisms
*Amyloid precursor protein (APP)*	Increase or alter Aβ production; alter Aβ42/Aβ40 ratio; accumulate the pathogenic forms of Aβ	Increase tau phosphorylation	Induce spine loss; shift spine from mushroom to stubby shape; reduce synaptic proteins; synaptic degeneration	Profound immuno-metabolic perturbations; microglia activation and gliosis	Lipid dyshomeostasis	Abnormal mitochondrial dynamics	Reduce autophagy, mitophagy	Lysosomal dysfunction; neuronal apoptosis and necroptosis
*Presenilin 1 (PSEN1)*	Alter intramembranous cleavage of the Aβ by γ-secretase and lead to production of longer amyloidogenic Aβ peptides; increase Aβ42/Aβ40 ratio	Hyperphosphorylation of tau	Impair synaptic plasticity; regulate homeostatic synaptic scaling	Alter production of cytokines; immune dysfunction; increase oxidative stress and oxyradical production	Dysregulation of cholesterol metabolism; elevate cellular cholesterol level	Perturb calcium signaling in lymphocytes and increase mitochondrial superoxide production	–	Potentiate cell cycle arrest and apoptosis; sensitize neurons to excitotoxicity; reduce abundance of newborn neurons
*Presenilin 2 (PSEN2)*	Increase Aβ production; alter Aβ42/Aβ40 ratio	Hyperphosphorylation of tau	–	Disrupt innate immune homeostasis; curb the proinflammatory response in microglia	Increase levels of cholesterol, low-density lipoprotein and triglyceride; decrease level of high density lipoprotein	Alter endoplasmic reticulum (ER) to mitochondrial calcium signaling	Alter many autophagy-related proteins demonstrating a buildup of autophagosomes	–
*Apolipoprotein E (APOE)ε4*	Promote aberrant Aβ deposition; influence APP processing and stimulate Aβ production; promote the shift of soluble Aβ into insoluble Aβ fibrils; reduce Aβ clearance	Promote the aberrant hyperphosphorylation of tau by dysregulating tau kinases and phosphatases; enhance p-tau aggregation into tangles	Reduction in dendritic arborization, length and spines density	Microglia and astrocyte activation; proinflammatory cytokines release; higher levels of oxidative stress	Abnormal lipid metabolism transport and homeostasis; less lipid delivery and clearance; elevated lipid peroxidation	Lower mitochondrial cytochrome oxidase activity	–	Increase blood–brain barrier permeability; gamma-amino butyric acid positive interneuron loss; glucose hypometabolism
*Triggering receptor expressed on myeloid cells 2 (TREM2)*	Sustain the microglial response to Aβ plaque-induced pathology; augment Aβ accumulation	Promoting the spreading of pathogenic tau	Impair synapse elimination	Alter microglial function including proliferation, survival, clustering, and phagocytosis	–	–	Anomalous autophagy in microglia	Neurodegeneration; enhance excitatory neurotransmission and reduce long-range functional connectivity
*SORL1*	Alter levels of Aβ and interfere with APP trafficking	Alter interaction with tau protein, associated with increased tau concentrations	–	–	–	Alter energy production, mRNA translation and mammalian target of rapamycin complex 1 signaling pathway	Autophagy dysfunction	Alter endosomal recycling pathway in neurons; defect in lysosome function
*Adenosine triphosphate-binding cassette transporter subfamily A member 7 (ABCA7)*	Alter APP processing and inhibit Aβ secretion	–	Anomalies in synaptic plasticity in lateral entorhinal cortex but not hippocampus	Alter microglial responses to acute inflammatory challenges; alter phagocytic activity in macrophages	Alter homeostasis of phospholipids and cholesterol	–	–	Decrease clearance of α-Synuclein; induce protein mislocalization

### Other Risk Genes

The confirmed genetic risk variants from SAD showed enrichment in FAD as well, but the risk scores were not statistically significant probably due to the small sample size (Reyes-Dumeyer et al. 2022). Some rare protein-damaging variants in *TREM2, SORL1* and *ABCA7* do have moderate-to-high effect, and cause FAD in an autosomal dominant nature, as described above, but most of them were present as singletons (Campion et al. 2019; Scheltens et al. 2021). There is disagreement about whether these loci reached genome-wide significance in association with AD, due to the differences in the criteria and number of subjects included, different analysis methods and research strategies (Campion et al. 2019; Scheltens et al. 2021; Reyes-Dumeyer et al. 2022). Functional annotation of these risk loci indicates that, next to Aβ metabolism, the modulation of the immune response, cholesterol, lipid dysfunction, endocytosis, and vascular factors play a role in the development of AD (Di Marco et al. 2015; Van Cauwenberghe et al. 2016; Naj et al. 2017; Bennett et al. 2018; Verheijen et al. 2018). The exact functional consequences of additional missense variants as well as corresponding levels of AD risk remain to be determined.

## Mechanisms of FAD vs. SAD

In general, FAD and SAD share common mechanisms, such as toxicity of Aβ and hyperphosphorylation of tau, oxidative stress, neuroinflammation, and autophagy dysfunction (Wang et al. 2014; Manoharan et al. 2016; Kodamullil et al. 2017; Li et al. 2017; Moloudizargari et al. 2017; Sawikr et al. 2017; Tönnies et al. 2017; Wu et al. 2017; Chen 2018; Kaur et al. 2019; Lu et al. 2019; Paroni et al. 2019). The most common mechanism is about Aβ. SAD and FAD both exhibit abundant deposition of Aβ peptides within brain cells, the extracellular space of the brain parenchyma, and the walls of the cerebral vasculature (Roher et al. 2016). Interestingly, AβPP levels in both *PSEN*-FAD and SAD remained within the limits of normal confidence established by non-demented, age-matched individuals (Roher et al. 2013). A study revealed that perturbations of intraneuronal signaling pathways comprise a common mechanistic denominator in both FAD and SAD, and such alterations lead to increases in AβO formation and phosphorylation of tau (Van Dooren et al. 2014). In addition, biomarker changes for FAD, in many but not all cases, appear to be similar to those for SAD (Lista et al. 2015).

Although sharing some common mechanisms, there are also differences between FAD and SAD. The familial form is due to mutations in pathogenic genes, while many genetic and environmental factors as well as unknown factors may contribute to determining the SAD form (Frisoni et al. 2022). FAD patients usually have an earlier age of onset and longer course than SAD patients (Armstrong 2014). FAD has more severe Aβ load and tau pathology, an earlier and quicker development of NFT, faster neuronal demise, and a diverse spectrum of distinctive neuropathological findings in the gray matter, including unusual 'cotton wool' amyloid plaques, Lewy bodies, Pick bodies, and ectopic neurons as well as white matter changes with atypical clusters of amyloid plaques and a variable degree of microhemorrhages (Gomez-Isla et al. 1999; Maarouf et al. 2008; Frisoni et al. 2022). Other co-morbidities like cerebrovascular disease, argyrophilic grain disease and hippocampal sclerosis were present in SAD but not in FAD (Cairns et al. 2015). In FAD, Aβ deposits are linked to increased synthesis or overproduction of Aβ peptides, while in SAD, Aβ accumulation may be the result of chronic AβPP/Aβ overproduction and limited degradation/clearance (Meraz-Rios et al. 2014; Roher et al. 2016). GWAS in SAD population showed that most of the risk genes affected the production and clearance of Aβ (Bertram et al. 2007). Increased Aβ42/43 production does not occur in most SAD cases (Ray et al. 1998). A study revealed that Notch1, Erb-B4, neurexin, neurofilament-L, neurofilament-M, α-tubulin, β-tubulin, dynein, and tau were substantially decreased in *PSEN*-FAD relative to SAD, while glial fibrillary acidic protein and neuroligin were increased (Roher et al. 2013). Equating SAD and *PSEN*-FAD only on the bases of their amyloid and NFT deposits hampered a better understanding of their pathogenesis and pathophysiology (Roher et al. 2016). Another study found that type I filaments were mostly in the brains of individuals with SAD, and type II filaments were found in individuals with FAD and other conditions (Yang et al. 2022). In FAD, the lifetime risk of dementia is very high, nearly 100% (Bateman et al. 2011), while in SAD, the percentage is lower, about 22%–95% in *APOE ε4*-related AD and 7%–35% in *APOE ε4*-unrelated AD (Genin et al. 2011; Reiman et al. 2020). More differentiating mechanisms should be studied in the future.

## AD Gene Therapy

Based on different genetic phenotypes of AD, vast avenues for gene therapy interventions are opened, aiming to tackle the disease at its source, mostly a faulty DNA/gene/protein, to repair it and allow the cells to fix the problem. Gene therapy involves inserting new genetic material into living cells using viruses. A deep understanding of the neuropathology of AD has also led to the development of numerous viral-mediated gene-transfer approaches (Khan et al. 2020; Mendell et al. 2021).

### Preclinical Studies of AD Gene Therapy

In rodent lesion models for AD, human neural stem cells (NSC) were used in place of fibroblasts to deliver nerve growth factor (NGF), which improved cognitive function (Wu et al. 2008; Lee et al. 2012). NSC's BDNF basal production and genetically modified NSC also showed efficacy in AD transgenic mouse models (Blurton-Jones et al. 2009; Wu et al. 2016). *Fibroblast growth factor2 (FGF2)* gene delivery via adeno-associated viruses serotype 2/1 hybrid (AAV2/1) could enhance neurogenesis and hippocampal Aβ clearance in AD mouse model, putting forward its usage as an alternative in AD therapy (Kiyota et al. 2011). Modified NSC-producing neprilysin led to improvement in synaptic density, and alleviated AD pathology in transgenic mice (Blurton-Jones et al. 2014). Mesenchymal stem cells (MSC) transplantation and miRNA-937 overexpression in MSC also showed efficacy on cognitive capabilities in AD mouse models (Tanna et al. 2014; Liu et al. 2015; Naaldijk et al. 2017; Parambi et al. 2022). In a recent preclinical study, by deleting a gene called Bax in FAD mice, the survival rate of stem cells was increased, leading to more neurons mature in hippocampus, such targeted augmentation of neurogenesis restored new neurons number in the engram, the dendritic spine density and the transcription signature ultimately led to the rescue of memory (Mishra et al. 2022a).

In addition, one preclinical study showed that peripheral administration of antisense oligonucleotides (ASO) targeting AβPP reversed AβPP and low-density lipoprotein-related protein-1 (LRP-1) overexpression in the aged SAMP8 mouse of AD (Erickson et al. 2012). Treatment of AD mice with a single dose of ASO that increases exon 19 splicing corrected *APOE receptor 2* splicing for up to six months and improved synaptic function and learning and memory (Hinrich et al. 2016). Using an ASO to reduce APOE expression in the brains of APP/PSEN1-21 mice prior to plaque deposition strongly affected the initiation of Aβ pathology, while lowering APOE after Aβ seeding modulated plaque size and toxicity (Huynh et al. 2017). In another study, delivering the *peroxisome proliferator-activated receptor gamma coactivator 1 alpha* (*PGC1-α*) gene using a modified virus to mice brain cells reduced the development of AD, and the treated mice showed better memory, no loss of brain cells in the hippocampus and had very few amyloid plaques after four months of injection (Katsouri et al. 2016).

### Clinical Trials of AD Gene Therapy

Some approaches have entered clinical trials. One approach is the delivery of NGF, which is hypothesized to promote the survival of cholinergic neurons (Fischer et al. 1987). Intracerebral delivery of NGF using recombinant AAV to the basal forebrain of patients with mild to moderate AD showed safety and well tolerance (Rafii et al. 2014). However, efficacy endpoints were not met in the subsequent phase 2 study (Rafii et al. 2018). Another study subjected 10 patients with early AD with NGF gene ex vivo or in vivo, and the researchers found a positive response of neurons showing cell hypertrophy, axonal sprouting, and activation of functional markers, and the sprouting induced by NGF persisted for 10 years after gene transfer and appeared safe (Tuszynski et al. 2015). Thus, the study needs confirmation of precise gene targeting. In a recent breakthrough, scientists found a genetic snipping technique which can be used to turn *APOE4*, the gene that is responsible to cause Aβ proteins in the brain, into *APOE3* (Khan et al. 2020). Taken together, gene editing and transferrin and penetratin-tagged liposomal nanoparticles might be the answer to solve gene format and dosage issue (Dos Santos Rodrigues et al. 2019; Williams et al. 2020).

In addition to the above clinical studies of gene therapy, there are more evidence showing that AD variants are used for therapy. For example, since *PSEN1* E280A variant was reported from Colombia, it has been further translated into a phase 2 clinical trial (ClinicalTrials.gov Identifier: NCT01998841), examining the effectiveness and safety of the drug crenezumab in presymptomatic participants carrying this variant in autosomal-dominant AD (ADAD) population (Tariot et al. 2018). Lecanemab, E2801, Gantenerumab and Solanezumab are used in phase 2/3 clinical trials in individuals with mutations causing dominantly inherited AD from the DIAN population (NCT01760005, NCT05269394, NCT05552157). LX1001, a serotype rh.10 AAV gene transfer vector expressing the cDNA coding for human *APOE2* is used in a phase 1/2 clinical trial in individuals with *APOE4* homozygote AD (NCT03634007).

### Future Perspective of AD Gene Therapy

Gene therapy is the recent addition as therapeutic agents for AD, however, they are yet to be clinically approved. Because different genetic subtypes of AD show different symptoms or disease courses, whether there is overlap and conversion between different subtypes requires further research. For example, the synaptic loss is more obvious in EOAD, which can affect acetylcholine, norepinephrine γ-aminobutyric acid and humoral protein levels (Bigio et al. 2002). Therefore, the clinical trials of gene therapy for EOAD should intervene earlier than the usual prototype disease, and considering the polygenic nature of most AD cases, the combined treatment of multiple neurotransmitters as well as multiple genes may be more effective in improving symptoms than the single-target cholinergic drugs. Attention should be paid to genetic phenotypes when conducting clinical trials of AD gene therapy, which can enable researchers to design experiments more accurately, select appropriate subjects, and obtain reliable efficacy and safety results.

In addition to genetic phenotypes, AD gene therapy should also consider gene-environment interactions, since various environmental factors contribute to the complex etiology of AD. Studies in both animal models and humans have shown that environmental AD risk factors, such as diet, lifestyle, alcohol, smoking and pollutants, can induce epigenetic modifications of key AD-related genes and pathways namely oxidative stress (Migliore et al. 2022). Furthermore, among the environmental risk factors, many are preventable, such as depression, social isolation, low educational levels, hearing impairment, physical inactivity, smoking, obesity, hypertension, diabetes, alcohol abuse, and air pollution (Jia et al. 2020a; Livingston et al. 2020). As a result, the environmental risk factors should also be taken into account when conducting gene therapy clinical trials, especially for LOAD patients and patients from different environmental settings. For example, selecting patients with similar environmental factors, or setting environmental factors as covariates when conducting multi-center gene therapy clinical trials.

## Conclusions

Several genes contributed to the genetic pathogenesis and high risk of FAD. Different pathogenic genes showed various phenotypes and underling molecular mechanisms, some of which are shared with SAD, while some are unique to specific mutations. Future gene therapy for AD should pay more attention to the genetic phenotypes and adopt more precise and individualized treatment strategies in designing clinical trials.

## Data Availability

Not applicable.

## Abbreviations

AAV2/1: Adeno-associated viruses serotype 2/1 hybrid
ABCA7: Adenosine triphosphate-binding cassette transporter subfamily A member 7
AD: Alzheimer’s disease
ADAD: Autosomal-dominant Alzheimer’s disease
AICD: APP intracellular domain
APOE: Apolipoprotein E
APP: Amyloid precursor protein
ASO: Antisense oligonucleotides
AβO: Aβ oligomers
AβPP: Amyloid-β protein precursor
BDNF: Brain-derived neurotrophic factor
BIN1: Bridging integrator 1
CD33: Cluster of differentiation 33
CFAN: Chinese familial Alzheimer’s network
CLU: Clusterin
CR1: Complement receptor 1
CREB: Cyclic adenosine monophosphate response element binding protein
DAPK1: Death-associated protein kinase 1
DIAN: Dominantly Inherited Alzheimer network
EOAD: Early onset AD
ER: Endoplasmic reticulum
FAD: Familial AD
FGF2: Fibroblast growth factor 2
GLUA1: Glutamate receptor subunit α-Amino-3-hydroxy-5-methyl-4-isoxazolepropionic acid
GWAS: Genome-wide association studies
IL: Interleukin
LOAD: Late-onset AD
LRP-1: Low-density lipoprotein-related protein-1
MAP2: Microtubule-associated protein 2
MSC: Mesenchymal stem cells
NFT: Neurofibrillary tangles
NGF: Nerve growth factor
NKG2D: Natural killer group 2 member D
NMDA: N-methyl-d-aspartate
NSC: Neural stem cells
PGC1-α: Peroxisome proliferator-activated receptor gamma coactivator 1 alpha
PKA: Protein kinase
PSEN1: Presenilin 1
PSEN2: Presenilin 2
SAD: Sporadic AD
SM: Sphingomyelin
SORL1: Sortilin-related receptor 1
SORLA: Sorting-related receptor with A-type repeats
TNFα: Tumor necrosis factor-α
TREM2: Triggering receptor expressed on myeloid cells 2
WES: Whole exome sequencing
WGS: Whole genome sequencing
